# Enhanced bioethanol production from wheat straw hemicellulose by mutant strains of pentose fermenting organisms *Pichia stipitis* and *Candida shehatae*

**DOI:** 10.1186/s40064-016-3222-1

**Published:** 2016-09-13

**Authors:** Sravanthi Koti, Sai Prashanthi Govumoni, Jahnavi Gentela, L. Venkateswar Rao

**Affiliations:** Department of Microbiology, Osmania University, Hyderabad, Telangana state 500 007 India

**Keywords:** Wheat straw, Hemicellulose, Mutant, Pentose, Yeast, Fermentation

## Abstract

The main aim of the present study was to mutate yeast strains, *Pichia stipitis* NCIM 3498 and *Candida shehatae* NCIM 3501 and assess the mutant’s ability to utilize, ferment wheat straw hemicellulose with enhanced ethanol yield. The organisms were subjected to random mutagenesis using physical (ultraviolet radiation) and chemical (ethidium bromide) mutagens. The mutant and wild strains were used to ferment the hemicellulosic hydrolysates of wheat straw obtained by 2 % dilute sulphuric acid and enzymatic hydrolysis by crude xylanase separately. Among all the mutant strains, PSUV9 and CSEB7 showed enhanced ethanol production (12.15 ± 0.57, 9.55 ± 0.47 g/L and yield 0.450 ± 0.009, 0.440 ± 0.001 g/g) as compared to the wild strains (8.28 ± 0.54, 7.92 ± 0.89 g/L and yield 0.380 ± 0.006 and 0.370 ± 0.002 g/g) in both the hydrolysates. The mutant strains were also checked for their consistency in ethanol production and found stable for 19 cycles in hemicellulosic hydrolysates of wheat straw. A novel element in the present study was introduction of chemical mutagenesis in wild type as well as UV induced mutants. This combination of treatments i.e., UV followed by chemical mutagenesis was practically successful.

## Background

Fossil fuel reserves are limited, and their current oxidation rate is a major global environmental concern with complex and severe climatic impacts (Stephenson et al. 2011). The increase in agro-industrial activity has led to the widespread accumulation of large quantities of lignocellulosic residues from wood, forestry, herbaceous, agricultural, solid, and various industrial wastes (José et al. 2010). These residues are collectively termed “biomass” and can be converted into ethanol fuel. Although bioethanol production has improved greatly by new technologies, further investigation is required to overcome the many remaining challenges (Yan and Shuzo 2006).

The production of ethanol from lignocellulosic biomass involves three major processes: pretreatment, hydrolysis, and fermentation. Pretreatment is required to alter the size, structure, and chemical composition of the biomass to facilitate rapid and efficient hydrolysis (Chang and Holtzapple 2000). Recent advances in pretreatment technology have the potential to improve the efficiency and reduce the cost of ethanol production (Mosier et al. 2003). Dilute acid pretreatment has become a state-of-the-art technology for the conversion of hemicellulose from any lignocellulosic biomass source into sugars. The fermentable sugars obtained via hydrolysis could subsequently be fermented into ethanol by ethanol-producing microorganisms (Lee et al. 1999).

Hemicellulose, a branched polymer composed of pentose and hexose sugars, can be hydrolyzed by hemicellulases or acids to release its monomeric sugars. Xylose and arabinose generally constitute a significant fraction of lignocellulosic biomass; therefore, their utilization is essential for a feasible bioethanol production process (Aristidou and Penttila 2000; Bothast et al. 2002; Koti et al. 2012; Sues et al. 2005). The microbial strain selected for the fermentation of pentose sugars has a large effect on ethanol yield (Anuj et al. 2011). Therefore, naturally xylose-fermenting yeasts such as *Candida shehatae* and *Pichia stipitis* have been widely studied because of their ability to ferment xylose into ethanol (Borbala et al. 2012). *Pichia stipitis* is considered a promising strain because it can ferment a wide range of sugars, including cellobiose (Nigam 2001). Furthermore, Candida species have been shown to ferment d-xylose to ethanol as the major product (Gong et al. 1983). The enhancement of microbial strains through mutation or gene cloning has gained attention in the commercial fermentation industry as a means to increase ethanol yields. Mutational enhancement of microorganisms is an old technique; however, use of this approach has resulted in improved ethanol yields at the laboratory level in several studies (Anuj et al. 2011).

In the present study, efforts were made to improve the pentose fermenting yeast strains by mutations using physical (UV irradiation) and chemical (ethidium bromide treatment) mutagens and selected mutant strains were assessed for their ability to produce enhanced yields of ethanol from wheat straw.

## Methods

### Substrate and yeast strains

The wheat straw used in the present study was obtained from Medak, Telangana state, India and the type used was *Triticum dicoccum*. The straw was dried at 60 °C in a hot air oven until the constant weight was obtained and processed in a laboratory pulverizer, seived to attain a particle size between 1–3 mm. In order to avoid analytical interferences, the substrate was washed before the hydrolysis with tap water to make it free from dust and dried at 65 ± 0.5 °C for overnight. The cellulose, lignin and hemicellulosic fractions of pulverized wheat straw were determined according to ASTM (2007) method.

Yeast strains of *Pichia stipitis* NCIM 3498, *Candida sheatae* NCIM 3501 were obtained from National Chemical Laboratory, Pune, India and maintained on MGYPX agar (g/L: peptone, 10; yeast extract, 10; d-glucose, 20; xylose, 5; agar, 20).

### Mutagenesis of yeast strains

#### UV mutagenesis

UV mutagenesis was carried out according to the method of Winston and Ausubel (Winston and Ausube 1990). Overnight grown cultures of *Pichia stipitis* and *Candida sheatae* (5 mL) were washed and re-suspended in 0.1 M phosphate buffer (pH 5.4) in order to achieve 10^8^ cells per ml. The above cell suspension (2 mL) was placed in a sterile Petri dish and exposed to UV rays at a distance of 20 cm. At regular intervals (15, 30, 45, 60 min), the samples were collected and serially diluted to have 200–300 viable cells in each plate. Then the samples were plated on MGYPX agar medium and incubated at 28 °C for 48 h.

#### Chemical mutagenesis

The wild strains and UV induced mutants were grown for overnight in MGYPX medium and the cells after incubation were washed and suspended in 0.1 M phosphate buffer (pH 5.4). A stock of 0.1 mg/mL ethidium bromide was prepared and from this 1 mL of ethidium bromide was added to 9 mL of phosphate buffer containing yeast cells. After specific time intervals of 30, 60, 90, 120, 150 and 180 min of incubation, the cell suspensions were centrifuged at 3000 rpm for 5 min to remove the traces of mutagen. Cells were plated on MGYPX agar plates and incubated at 28 °C (Joanna and Ewelina 2003).

### Enzyme assay

Xylanase assay was performed using 1 % (w/v) oat spelt xylan as substrate (Bailey et al. 1992). One unit (IU) of enzyme activity is defined as the amount of enzyme that produces 1 μmol of xylose in the reaction mixture per minute under the assay conditions used.

### Preparation of the wheat straw hemicellulosic hydrolysates

#### Pretreatment of wheat straw with NaOH

Wheat straw (250 g) was pretreated using 1.5 % (w/v) NaOH for 2 h at 100 °C with a liquid to solid ratio of 10:1 (Sai Prashanthi et al. 2013). The substrate was squeezed, washed and neutralized with tap water. Delignified filtrate obtained was analyzed for sugars and phenolic inhibitors (Miller 1959; Singleton et al. 1965).

#### Acid hydrolysis

Alkali pretreated wheat straw (50 g) was hydrolyzed at 121 °C with 2 % (v/v) sulfuric acid for 60 min, with an initial liquid to solid ratio of 10:1. The suspension was then squeezed to remove the unhydrolysed residue. The hydrolysate obtained was neutralized, detoxified and analyzed for sugars (Nigam 2001).

#### Enzyme production and enzymatic hydrolysis

The production media contained 50 g of corn cobs moistened with 50 mL mineral solution containing (g/L): KH_2_PO_4_, 28; (NH_4_)_2_ SO_4_, 19.6; Urea, 4.2; MgSO_4_·7H_2_0, 4.2; CoCl_2_, 4.2; FeSO_4_·7H_2_0, 0.07; MnSO_4_·7H_2_0, 0.021; ZnSO_4_·7H_2_0, 0.019; CaC1_2_, 0.028; yeast extract, 7 and glucose, 15; pH 5.0 ± 0.2. The media were inoculated with 10 ml of inoculum having 10^6^ spores/mL collected from 72 h grown culture of *Trichoderma asperellum* (Genebank accession number-KP965729). Inoculated production media were incubated under static conditions at 28 ± 2 °C and enzyme production was checked after every 24 h for 5 days. Enzyme was extracted with 500 mL of 0.05 M sodium acetate buffer on a rotary shaker at 150 rpm for 30 min. The content was filtered through muslin cloth and the filtrate was used as the enzyme source and utilized for enzymatic saccharification at a dosage of 0.25 mL (250 IU/mL, pH 4.8) per gram of alkali pretreated wheat straw (50 g) and incubated at 50 ± 0.5 °C, 150 rpm for 48 h. After incubation, the hydrolysate was seperated by filtration, supplemented with nutrients, sterilized and fermented to ethanol by wild and mutant strains.

### Inoculum and fermentation media

The inoculum was developed by inoculating the wild type and mutant strains using media containing (g/L: xylose, 25; glucose, 5; yeast extract 5; malt extract, 5; peptone, 5; pH, 5.5) and incubated at 28 °C on a rotatory shaker at 200 rpm. The optical density (OD) of inoculum cultures was determined at the wavelength of 600 nm using a Systronics 117 UV–Vis spectrophotometer. Each fermentation media was inoculated with 1.2 × 10^8^ cells based on the conversion factor of 0.50 OD being equal to 1 × 10^7^ cells. The fermentation media used were 2 % acid hydrolysate and enzymatic hydrolysate consisted of 39.3 and 35.73 g/L of sugars respectively. Both the hydrolysates were supplemented with (g/L); yeast extract, 2; (NH_4_)_2_SO_4_, 1; K_2_HPO_4_, 0.5; peptone, 1; MgSO_4_, 0.5; MnSO_4_, 0.5.

### Ethanol fermentation

Ethanol fermentation was performed at 28 °C using wheat straw acid and enzymatic hydrolysates. As the fermentation was carried out in triplicates, average and standard deviations were calculated. Samples were collected at regular time intervals (12 h), centrifuged and supernatants obtained were examined for concentration of reducing sugars and ethanol.

### Analytical methods

The amount of reducing sugars liberated was determined using the Dinitrosalicylic acid method with xylose/glucose as standard (Miller 1959). Ethanol concentration was analyzed by Gas chromatography (GC) (Shimadzu 2010, Japan) using ZB Wax column (30 mm × 0·25 mm) with a flame ionization detector (FID). The analysis was performed according to NREL (National Renewable Energy Laboratory) procedure LAP #001. The conditions used were: 150 °C (isothermal), program run time: 5.5 min, ethanol retention time: 2.3 min and the carrier gas: nitrogen (16 kPa), injector temperature: 175 °C, detector temperature: 250 °C, flow rate: 40 ml/min, spilt ratio: 1/50, velocity of H_2_ flow: 60 ml/min and sample quantity: 1 μl. The supernatant was filtered by 0.22 μm cellulose acetate filters prior to GC analysis (Srilekha Yadav et al. 2011).

### Statistical analysis

To assess whether there was any significant difference in ethanol production between the wild type and mutant strains of *Pichia stipitis* and *Candida shehatae* in acid and enzymatic hydrolysates of wheat straw, a paired t test was performed using Graph pad software (Graph pad software, inc., La Jolla, CA 92037 USA).

## Results and discussion

### Compositional analysis of wheat straw

The cellulose, lignin and hemicellulosic fractions of pulverized wheat straw were determined according to ASTM (2007) method and reported in our previous study (Sai Prashanthi et al. 2013). The average percentages of acid soluble lignin, acid insoluble lignin, cellulose and hemicellulose were found to be 16 ± 1.15, 4.6 ± 0.26, 32.60 ± 0.37 and 24.7 ± 0.2 % respectively.

### UV and EtBr mutagenesis

After separate UV and chemical mutagenesis, combination of UV and chemical mutagenesis was performed and selection of large colonies (42 colonie; from each method 7 colonies) was done on ethanol-containing medium. All these mutants were screened for maximum ethanol production in synthetic fermentation medium. The highest ethanol producing isolates were picked up from the UV followed by EtBr treatment (PSUV9, CSUV4) and EtBr mutagenesis (PSEB5, CSEB7) exhibiting 0.1 to 1.0 %, survival. The mutants varied in cell size from parent and other mutants. Determination of ethanol produced by all the mutants revealed that only four mutants resulted in significant ethanol productivity in synthetic medium compared to their wild strains. In addition to the above four mutants, wild strains of *Pichia stipitis* NCIM 3498 and *Candida shehatae* NCIM 3501 were also investigated in this study as reference strains. The improvement of mutants in ethanol production (%) in fermentation medium II compared to the parent strains is summarized in Table 1.

**Table 1:** Improvement of the production of ethanol in fermentation medium II by treatment with two mutagenic agents

S. no.	Mutagenic treatment	Selected mutant strain	Ethanol production improved (%)
1	UV mutagenesis	PSUV9	46.73
		CSUV4	13.92
2	EtBr mutagenesis	PSEB5	11.39
		CSEB7	22.63

### Pretreatment of wheat straw with NaOH

Alkali pretreatment showed effective lignin solubilization of 70 % with minor cellulose and hemicellulose solubilization. Sugars and phenolics released during pretreatment were 0.83 and 17.28 g/L respectively.

### Dilute acid and enzymatic hydrolysis of wheat straw

The acid hydrolysate contained 6.8 g/L of phenolics, 0.334 ± 0.014 g/g reducing sugars (39.33 ± 1.33 g/L) with a practical conversion of 41.79 ± 1.62 % of the total carbohydrates present in the straw which indicates that some part of the cellulose was also hydrolyzed. After detoxification, the phenolic concentration in the hydrolysate was reduced to 0.2 g/L. The sugar yield in the present study is comparable to the results reported by Chandel et al. (2007) and Canilha et al. (2008). Chandel et al. reported 30.29 g/L of total reducing sugars using 2.5 % v/v HCl at 140 °C for 30 min with S:L ratio of 1:10 where as Canilha et al. reported 37 g/L of xylose by dilute acid hydrolysis of wheat straw.

During the course of enzymatic hydrolysis, a regular increase in sugar release was observed till 48 h and remained constant thereafter. The hydrolysate contained 0.285 ± 0.005 g/g sugars (35.73 ± 1.25 g/L) with a hydrolysis efficiency of 35.73 ± 1.25 % after 48 h of treatment. These results are in accordance with other published results (Junhua et al. 2011). Results of the acid and enzymatic digestibility are shown in Table 2.

**Table 2:** Concentration of sugars (g/L) in acid and enzymatic hydrolysates

S. no.	Type of hydrolysis	Time of incubation (h)	g/L	Saccharification (%)
1.	Enzymatic hydrolysis	18	16.67 ± 0.77	18.75 ± 0.56
		24	23 ± 0.55	28.75 ± 0.32
		48	35.73 ± 0.39	35.73 ± 0.65
2.	Dilute acid hydrolysis (2 %)	–	39.3 ± 0.46	41.79 ± 0.62

### Fermentation of wheat straw hydrolysates

#### Fermentation of acid hydrolysate

**Fig. 1:**
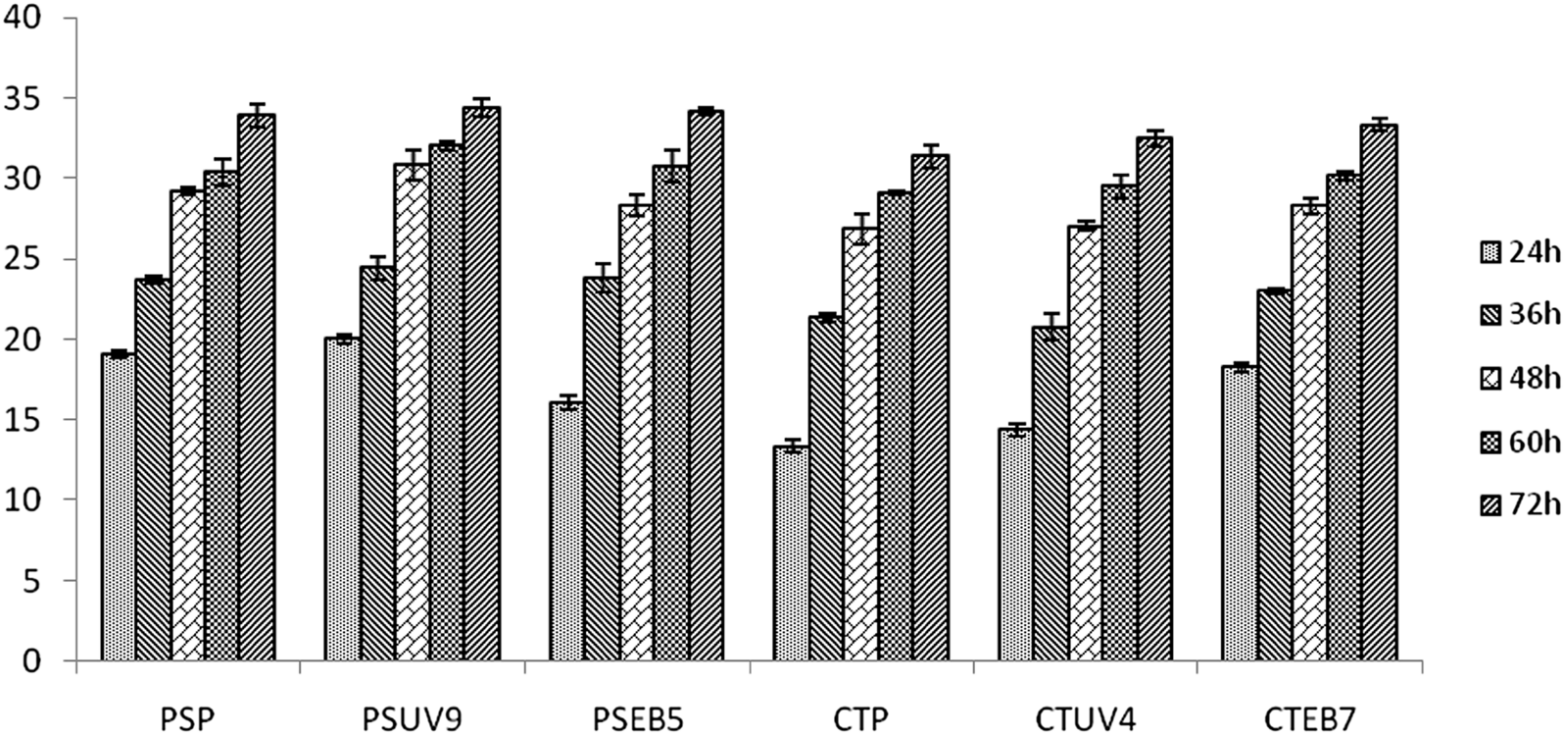
Total sugars utilization (g/L) in dilute acid hydrolysate by yeast strains at different time intervals

**Fig. 2:**
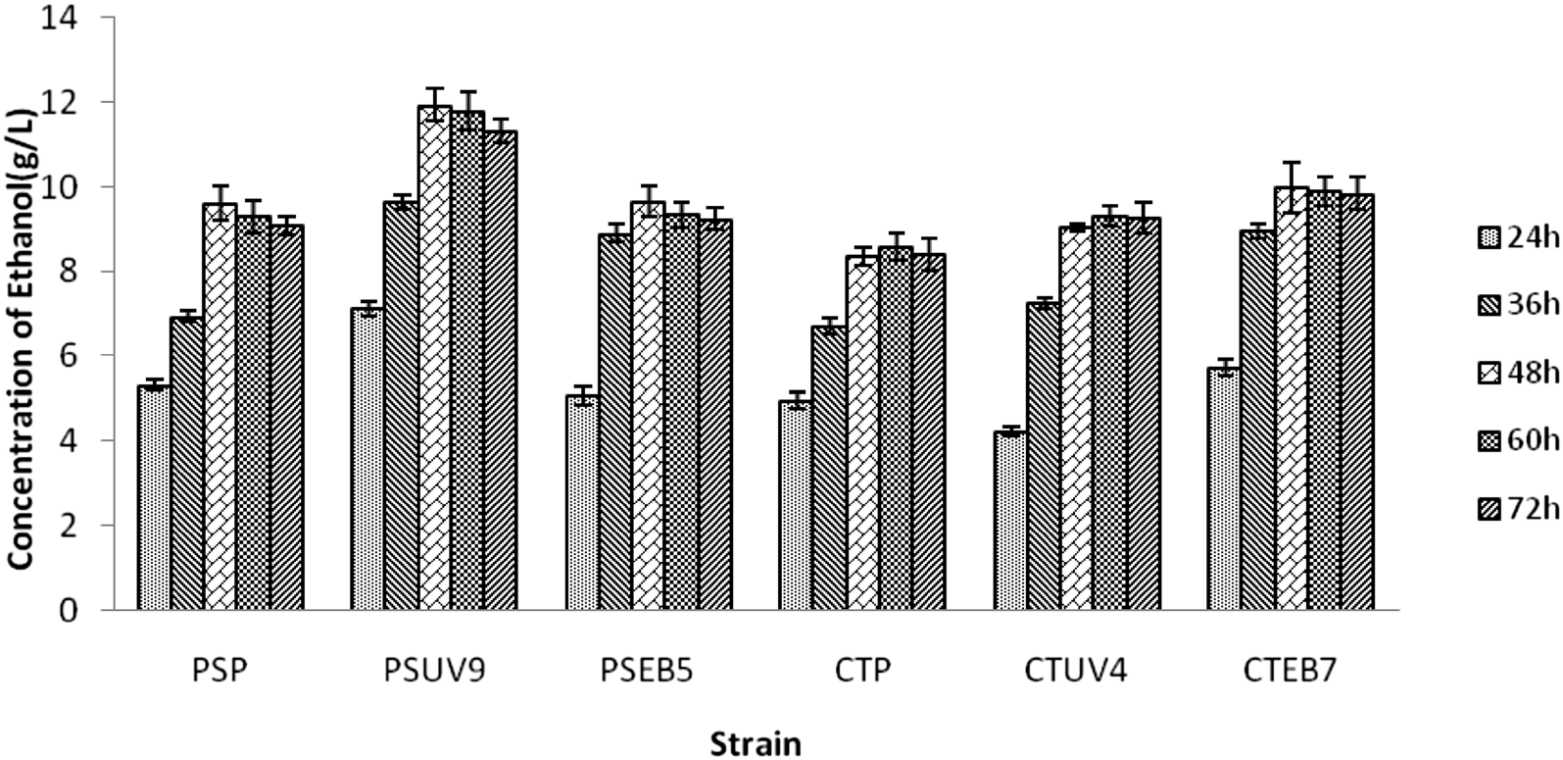
Concentration of ethanol (g/L) in dilute acid hydrolysate by yeast strains at different time intervals

**Fig. 3:**
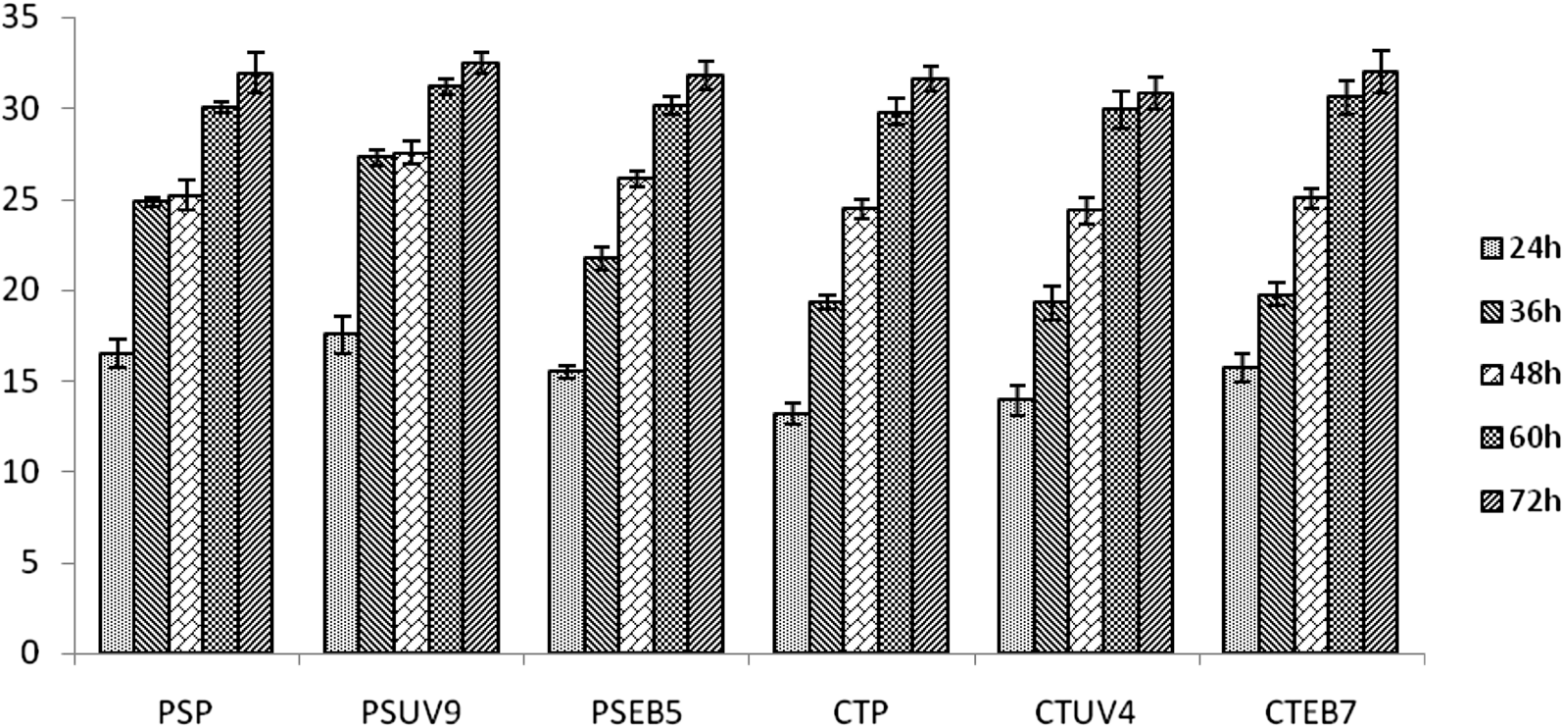
Total sugars utilization (g/L) in enzymatic hydrolysate by yeast strains at different time intervals

**Fig. 4:**
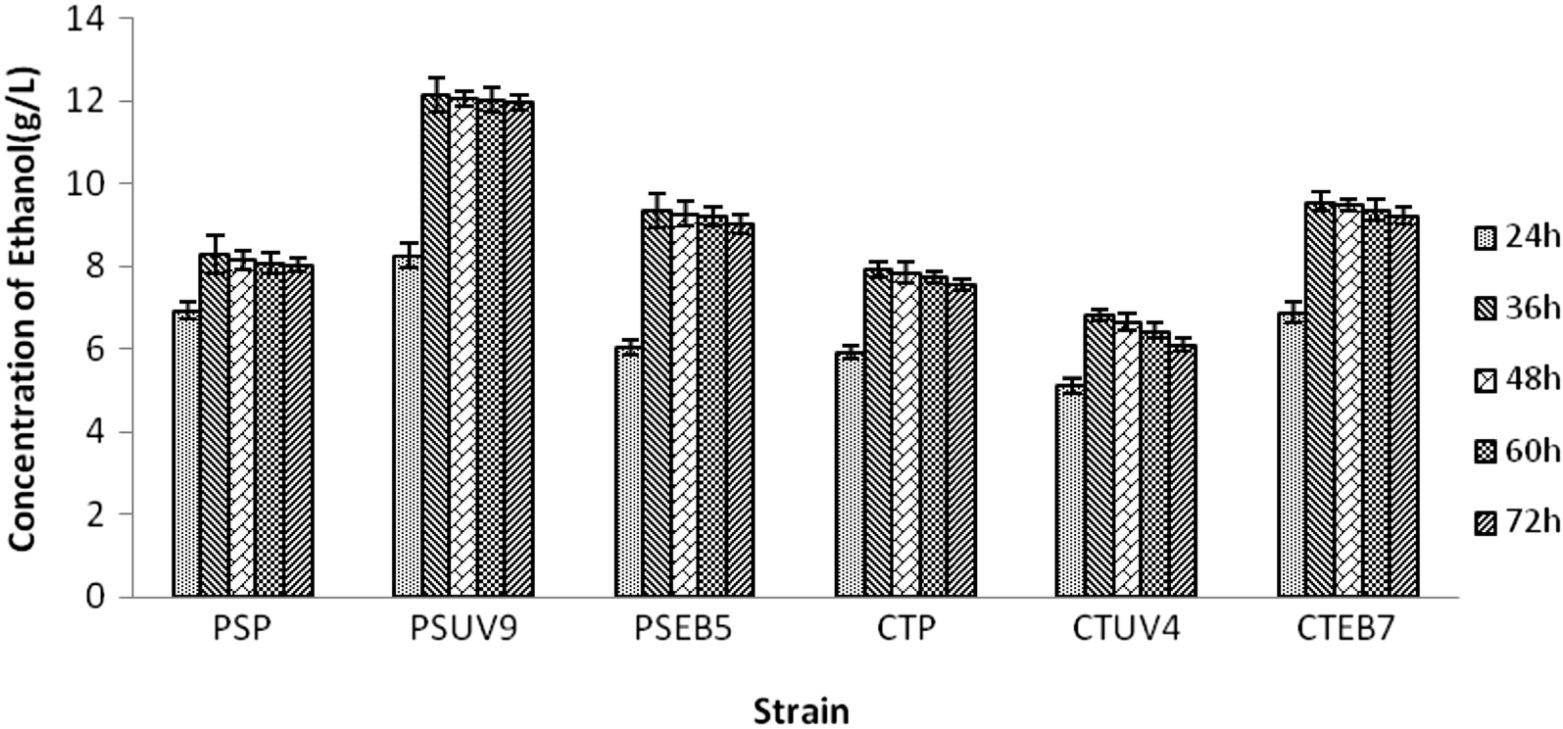
Concentration of ethanol (g/L) in enzymatic hydrolysate by yeast strains at different time intervals

The results of sugar consumption and ethanol production in fermentation studies are summarized in Figs. 1, 2, 3 and 4 respectively where as the kinetic parameters are summarized in Table 3. Among the four mutants of *Pichia stipitis* (PSUV9 and PSEB5) and *Candida shehatae* (CSUV4 and CSEB7), PSUV9 showed maximum ethanol production and fermentation efficiency (FE) (11.93 ± 0.38 g/L, yield 0.390 ± 0.008 g/g, FE 75.95 ± 0.26 %) followed by CSEB7 (9.98 ± 0.81 g/L, yield 0.350 ± 0.005 g/g, FE 69.24 ± 0.18 %) and PSEB5 (9.65 ± 0.74 g/L, yield 0.340 ± 0.006 g/g, FE 66.81 ± 0.12 %) in 2 % dilute acid hydrolysate (Fig. 2). Similar results were obtained by Eken-Sarakoglu and Arslan (2000) with mutant strain of *Pichia stipitis* using corncob hydrolysate. Shi et al. mutated *P. stipitis* CBS 6054 by disrupting the cytochrome c gene which has given 21 % higher ethanol yield (0.46 g/g sugar) than the parental strain (0.38 g/g sugar) from 8 % (w/v) xylose (Shi et al. 1999). Li generated an efficient mutant from *C. shehatae* ATCC 22984 by UV irradiation suggesting that the introduction of a mutation is effective for the improvement of ethanol from 0.39 to 0.42 g/g (Li et al. 2012). Among all the mutants, PSUV9 has given good yields of ethanol and this may be because of its ability of high sugar uptake, ethanol tolerance and inhibitor tolerance in the acid hydrolysate which was studied in the initial characterization studies of mutants. PSUV9 was able to grow after lag phase of 36 h at 1.5 g/L of vanillin, and 6 % of ethanol.

**Table 3:** Kinetic parameters for ethanol production from acid and enzymatic hydrolysates using the two best mutant strains (PSUV9, CSEB7) and parent strains (PSP, CSP) of *P*. *stipitis* NCIM-3498 and *Candida shehatae* NCIM 3501

Parameters	*Pichia stiptis*				*Candida shehatae*			
	Acid hydrolysate (48 h)		Enzymatic hydrolysate (36 h)		Acid hydrolysate (48 h)		Enzymatic hydrolysate (36 h)	
	Parent strain (PSP)	*Mutant strain* (PSUV9)	Parent strain (PSP)	*Mutant strain* (PSUV9)	Parent strain (CSP)	*Mutant strain* (CSEB7)	Parent strain (CSP)	*Mutant strain* (CSEB7)
Ethanol (g/L)	9.61 ± 0.39	11.93 ± 0.38	8.28 ± 0.54	12.15 ± 0.57	8.35 ± 0.36	9.98 ± 0.81	7.92 ± 0.89	9.55 ± 0.47
Sugar utilized (g/L)	29.20 ± 0.25	30.80 ± 0.93	21.84 ± 0.27	27.27 ± 0.42	26.85 ± 0.91	28.26 ± 0.52	21.35 ± 0.40	21.75 ± 0.64
Yield (g/g)	0.330 ± 0.008	0.390 ± 0.008	0.380 ± 0.006	0.450 ± 0.009	0.310 ± 0.007	0.350 ± 0.005	0.370 ± 0.002	0.440 ± 0.001
Productivity (g/L/h)	0.200 ± 0.015	0.240 ± 0.004	0.230 ± 0.005	0.330 ± 0.011	0.170 ± 0.007	0.200 ± 0.012	0.220 ± 0.007	0.260 ± 0.002
% conversion efficiency	64.53 ± 0.24	75.95 ± 0.26	74.34 ± 0.22	87.36 ± 0.37	60.98 ± 0.23	69.24 ± 0.18	72.74 ± 0.39	86.09 ± 0.31

In case of the wild strains, the maximum concentration of ethanol by *Pichia stipitis* (PSP) in dilute acid hydrolysate was found to be 9.61 ± 0.39 g/L equivalent to the yield 0.330 ± 0.008 g/g and fermentation efficiency of 64.530 ± 0.248 %. The yield obtained in this study for ethanol production by parent strain *P. stipitis* NCIM 3498 is comparable to the results reported earlier. Delgenes reported 0.25 g/g of ethanol yield from wheat straw dilute acid hydrolysate by *P. stipitis* (Delgenes et al. 1990). Roberto et al. and Nigam reported the same yield of ethanol i.e., 0.35 g/g from the sugarcane bagasse hydrolysate and *Eichhornia crassipes* by *P. stipitis* NRRL Y-7124 and *P*. *stipitis* CBS 5773 respectively (Roberto et al. 1991; Nigam 2002). In the present study, the parent strain of *Candida shehatae* (CSP) has produced 8.35 ± 0.36 g/L of ethanol which was equivalent to the yield of 0.310 ± 0.007 g/g and fermentation efficiency of 60.980 ± 0.237 %. Jing-Ping Ge also reported the same yield of ethanol i.e., 0.31 g/g with corncob hydrolysate using *Candida shehatae* ACCC 20335 (Jing-Ping et al. 2011). According to Tanimura, *Candida shehatae* strain ATY839 has produced 16.8 g/L i.e., 71.6 % of the maximum theoretical ethanol yield at 24 h (Ayumi et al. 2012).

#### Fermentation of enzymatic hydrolysate

The fermentability of wheat straw hemicellulosic enzymatic hydrolysate was also evaluated by wild and mutant strains of *Pichia stipitis* and *Candida shehatae*. Among the wild and mutant strains, PSUV9 showed highest concentration of ethanol (12.15 ± 0.57 g/L) after 36 h (Fig. 4). However, the ethanol productivity was almost static after 36 h of incubation. The fermentation efficiency was 87.36 ± 0.37 % and the resulting yield of ethanol was equivalent to 0.450 ± 0.009 g/g based on the total fermentable sugars (35.73 g/L) of the hydrolysate. Chandrasekhar Gajula produced a maximum ethanol yield of 0.44 g/g with *P. stipitis* NCIM 3498 respectively in batch fermentation conditions using ground nut shell enzyme hydrolysate (Chandrasekhar et al. 2011). We assume that the maximum yield of ethanol in this study is attributed to the occurrence and fermentation of pentoses and some amount of hexoses in the hydrolysate. Next to PSUV9, CSEB7 showed a maximum ethanol concentration of 9.55 ± 0.47 g/L which accounts for 0.440 ± 0.001 g/g ethanol yield and 86.09 ± 0.31 % of fermentation efficiency followed by PSEB4 with the ethanol concentration of 9.54 ± 0.61 g/L of ethanol which is equivalent to yield of 0.430 ± 0.002 g/g and efficiency of 84.28 ± 0.81 %.

The parent strains of *Pichia stipitis* and *Candida shehatae* have produced 8.28 ± 0.54 and 7.92 ± 0.89 g/L of ethanol which were equivalent to yields of 0.380 ± 0.006 and 0.370 ± 0.002 g/g respectively. It is clearly evident that the yield of ethanol was higher in enzymatic hydrolysate than acid hydrolysate using both wild and mutant strains and this poor fermentability of the acid hydrolysate might be due to the presence of some toxic components which remained after detoxification, affected the fermentation activity of the yeast.

### Statistical evaluation

The results of paired t test (Table 4) show that there is significant difference among all the pairs of wild and mutant strains. Since the pair 1(PSP&PSUV9) has more significance compared with the other pairs with *t* value (125.03) and P value (<0.0001), PSUV9 was found to be the best mutant among all the mutants.

**Table 4:** Statistical evaluation (paired samples test) of ethanol production (g/L) in acid and enzymatic hydrolysates of wheat straw by wild type mutants of *Pichia Stipitis* and *Candida shehatae*

	Paired differences (dependent sample *t* test)					
	95 % Confidence interval of the difference					
	Mean	Mean differences	SD	*t*	P value	Significant Y/N
Pair 1	5.31 7.12	1.81	0.02516	125.03	<0.0001	Y****
Pair 2	5.31 5.07	0.24	0.03214	12.572	0.0063	Y**
Pair 3	4.96 4.23	0.73	0.04509	28	0.0012	Y**
Pair 4	4.96 5.73	0.77	0.0450	29.064	0.0012	Y**
Pair 5	6.91 8.26	1.35	0.0208	112.88	0.0001	Y****
Pair 6	6.91 6.04	0.87	0.03214	46.150	0.0005	Y***
Pair 7	5.93 5.11	0.82	0.02081	67.674	0.0002	Y***
Pair 8	5.93 6.88	0.95	0.06658	25.580	0.00015	Y**

Pair 1: Ethanol production (PSP vs PSUV9) in dilute acid hydrolysate of wheat straw

Pair 2: Ethanol production (PSP vs PSEB5) in dilute acid hydrolysate of wheat straw

Pair 3: Ethanol production (CTP vs CTUV4) in dilute acid hydrolysate of wheat straw

Pair 4: Ethanol production (CTP vs CTEB7) in dilute acid hydrolysate of wheat straw

Pair 5: Ethanol production (PSP vs PSUV9) in enzymatic hydrolysate of wheat straw

Pair 6: Ethanol production (PSP vs PSEB5) in enzymatic hydrolysate of wheat straw

Pair 7: Ethanol production (CTP vs CTUV4) in enzymatic hydrolysate of wheat straw

Pair 8: Ethanol production (CTP vs CTEB7) in enzymatic hydrolysate of wheat straw

*Y/N* yes/no

****P ≤ 0.0001; ***0.0001 > P < 0.0009; **P > 0.0009

### The stability of the mutant *Pichia stipitis* PSUV9 and *Candida shehatae* CSEB7

**Fig. 5:**
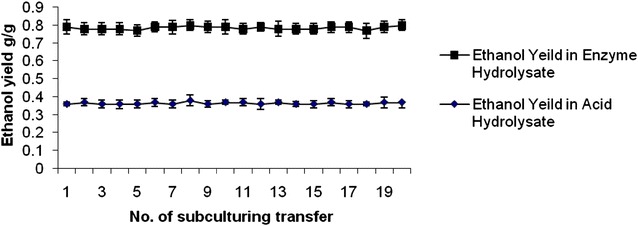
Stability of the production of ethanol by mutant *Pichia stiptis* PSUV9 in acid and enzymmatic hydrolysates

The stability of the mutants *Pichia stipitis* PSUV9 and *Candida shehatae* CSEB7 for increased ethanol production was determined by successive subculturing on MGYP plates for 19 generations (generation time of 48 h). After each subculture, the mutants were tested for their ability to produce consistent levels of ethanol in acid and enzymatic hydrolysates of wheat straw. The mutants maintained the consistent yields after 19 fermentaion cycles indicating that the mutation is stable (Fig. 5).

## Conclusions

Strain improvement by mutation is one of the best methods to increase the ethanol yield and in this case, we were able to obtain two strains capable of producing significantly higher ethanol yields than the wild strains. The mutant strains PSUV9 and CSEB7 showed higher ethanol production rates from wheat straw as compared to the wild strains. This research demonstrates the utility of random mutagenesis to generate advantageous strains of *P. stipitis* and *C. shehatae*. However, some significant improvements with regard to the two pentose fermenting yeast strains have been reached and these improvements offer new possibilities for further optimization.
